# They are not always a burden: Older people and child fostering in Uganda during the HIV epidemic

**DOI:** 10.1016/j.socscimed.2014.05.002

**Published:** 2014-07

**Authors:** Susan Kasedde, Aoife M. Doyle, Janet A. Seeley, David A. Ross

**Affiliations:** a MRC/UVRI Uganda Research Unit on AIDS, Uganda Virus Research Institute, P.O. Box 49, Entebbe, Uganda; b London School of Hygiene & Tropical Medicine, Keppel St, London WC1E7HT, UK; c UNICEF House, 3 United Nations Plaza, Office #1016, New York, NY 10017, USA

**Keywords:** Uganda, HIV, Child, Orphaned, Aged, Foster home care, Caregivers

## Abstract

This qualitative study examines the role of older people (60 years and above) in fostering decisions for orphans and non-orphans within extended families in a rural Ugandan community heavily affected by HIV. Fieldwork conducted in 2006 provided information on the influence of HIV on fostering decisions through 48 individual in-depth interviews and two group interviews with foster-children and family members to develop detailed case studies related to 13 fostered adolescents. The adolescents included five non-orphans and eight orphans (five were double orphans because they had lost both parents). Older people play a very important role in fostering decisions as potential foster-parents, advisers, mediators and gatekeepers. They have a high level of authority over the foster-children, who are regarded as important resources within the extended family. With fewer potential caregivers available because of HIV-related deaths, the responsibility for fostering orphans has often fallen to surviving older people. Fostering is used by older people and the child's extended family as a strategy to ensure the welfare of the foster-child. When the foster-parent is an older person, it is also used to ensure physical and emotional support for the older person themselves. Support from the extended family towards foster households is widely reported to have been reduced by HIV by diminishing resources that would otherwise have been made available to support foster care. New initiatives and investment are required to complement community and family resources within well-managed social protection and welfare programmes. To be effective, such programmes will require adequate investment in administrative capacity and monitoring. They must aim to strengthen families and, recognizing that resources are limited, should prioritize the community's poorest households, rather than specifically targeting households with orphans or other foster-children.

## Highlights

• Older people play key roles in fostering decisions as potential foster parents, advisers, mediators and gatekeepers.
• Authority: during the fostering process older people advise on, and sometimes exercise control over, family resources.
• Older foster parents rely primarily on the foster children and the local community to support them in times of need.
• Reciprocity: fostering by grandparents is used to ensure the welfare of the grandparent as well as the foster child.
• HIV has reduced extended family support to fostering households, but stigma has not changed the fostering process greatly.

## 1 Introduction

Throughout many parts of sub-Saharan Africa, fostering, the provision of parental care and nurturing to a child by an adult who is not the child's natural or adoptive parent, has been and remains a widespread practice (Goody, 1982; Roscoe, 1965; Serra, 2009). Fostering serves multiple goals: education, care and socialisation of children, securing of economic and political benefits for families through reciprocity for children's services, and, providing a critical safety net for children (Bledsoe and Isiugo-Abanihe, 1989; Kamali et al., 1996; Madhavan, 2004; Mathambo and Gibbs, 2009; Nyambedha et al., 2001, 2003; Nyamukapa and Gregson, 2005; Page, 1989; Schenk et al., 2010; Zaba et al., 2004). The Human Immunodeficiency Virus (HIV) epidemic has had a profound impact on the many children who have lost one or both parents to the infection (Dalen et al., 2009; UNAIDS et al., 2004). While statistics on the number of children fostered before the epidemic are difficult to find, it is apparent that the number of children in foster care has increased because of the loss of their parents and other carers because of HIV. The 2011 Uganda Demographic and Health Survey reported that 19% of children <18 years lived in households without their parents, 68% of whom were non-orphans. Higher rates of fostering are seen among older children, girls and among paternal relatives (Table 1) (Uganda Bureau of Statistics (UBOS) and ICF International Inc, 2012). According to the 2009/10 Ugandan National Household Survey (Uganda Bureau of Statistics, 2010), 18% of households had taken in at least one orphan. Adult HIV prevalence in Uganda in 2011 was 7.3% (Ministry of Health, 2012).

While early literature on fostering in Africa focused on fostering as the sharing of parenting roles, it largely remained silent on the identity of the foster carers except to say that this depended on the motive for fostering (for example, whether the fostering was for vocational training purposes or for nurturing of a very young child) and on the kinship structure of the society (Goody, 1982; Page, 1989; Roscoe, 1965). In more recent literature, the role of older people and particularly of grandparents in fostering has become more apparent (Ardington and Leibbrandt, 2010; Cohen and Menken, 2006; Ice et al., 2010; Mugisha et al., 2013; Oleke et al., 2005; Seeley et al., 2009; Urassa et al., 1997). A review of national household survey data from 13 sub-Saharan African countries found that grandparents were common foster-parents, caring for between 24% and 64% of these orphans (Monasch and Boerma, 2004). The image of elderly grandparents surrounded by grandchildren orphaned by AIDS has come to symbolize the community-based challenge of care for orphans and vulnerable children (Ankrah, 1993; Foster, 2000; Freeman and Nkomo, 2006; Madhavan, 2004; Ntozi and Mukiza-Gapere, 1995; Seeley et al., 1993) as well as the vulnerability of older people as they age without their own children to care for them (Ice et al., 2010; McKinnon et al., 2013; Wright et al., 2012). However, the reciprocal nature of the fostering arrangement, which can be particularly important for older caregivers, is often forgotten (Skovdal et al., 2009). Lesthaeghe (1981), in his work on fertility and family size, suggested that decisions on family size were affected significantly by considerations of the transaction or investment costs; in other words the balance between costs of childrearing and the estimated value of prospective benefits. The fostering arrangement benefits older foster-parents, providing greater social inclusion and recognition to companionship and assistance in the home, all of which are important, even in the absence of HIV (Schroder-Butterfill, 2004). Conversely, the older foster-parent provides a relatively stable home and an experienced carer for the child (Ansell and Young, 2004). Nevertheless, the wider kin network has been expected to support members, including the elderly, and caring for foster-children was not a pre-condition for receiving care in older age.

Among Ugandan ethnic groups with patrilineal kinship structures, children have usually been fostered within the father's extended family. This would particularly be the case if they were orphaned, as the children would automatically take on their father's lineage and thus “belong” to this line (Doyle, 2013; Page, 1989; Roscoe, 1965). However, fostering of very young children, and particularly orphans, by maternal relatives is often seen as appropriate, at least in the short-term (Roscoe, 1965).

Studies have suggested that in many parts of Africa, the traditional safety net of the extended family may have been overwhelmed by the dramatic rise in the number of orphans and children in need of care as a result of the HIV epidemic (Foster, 2000; International HIV/AIDS Alliance and HelpAge International, 2003; Monasch and Boerma, 2004; Nyambedha et al., 2001; Seeley et al., 1993). Decreased parental ability to oversee fostering and widowing, coupled with an increase in pressure on the paternal family due to the growing numbers of AIDS orphans has also, in some patrilineal societies, led to the increased involvement of maternal relatives and non-relatives in the fostering decision process (Nyamukapa and Gregson, 2005; Oleke et al., 2005). Researchers have also commented on the transformative impact that HIV has had on the roles of older people, notably on their increased primary roles in income generation for themselves and the children they live with, as well as primary caregivers in the absence of parents and other relatives in their early to mid-adult years, who would otherwise customarily play this caregiving role (Appleton, 2000; Wright et al., 2012).

This paper seeks to provide a greater understanding of how HIV affects the extended family and decisions related to child fostering in central Uganda. We investigate the role of older people in the fostering process and their role in the decisions on where fostered children go. In addition, we ask if HIV influences this process and decision-making. Understanding this process, the underlying motivations for fostering and the factors affecting successful decisions and outcomes for children provides a basis for the assessment of the needs of vulnerable households and potential strategies for intervention and support.

We have drawn on a life course approach (Elder, 1994) to inform our analysis. This approach makes use of four thematic areas: (1) Linked lives: How a person's life is embedded in social relationships with relatives, friends and neighbours across the life-span has a profound effect on their educational, occupational, and social welfare. (2) The social timing of important life events: When a person takes up a role (such as foster parent), how long the role lasts and the sequencing of roles all influence the course of a person's life and those connected to them. (3) Historical time: When someone is born exposes that person to constraints and opportunities which may be different from another person who was born in a different period. And finally, (4) Human agency: How much people can or cannot influence the course of their life and the lives of others through the choices and decisions they make is affected by the amount of independent decision-making power or authority they have, for example.

## 2 Methods

### 2.1 Study setting

This qualitative study was carried out in Kalungu District, central Uganda, an area occupied predominantly by the Baganda, Uganda's largest ethnic group. This region has long had high levels of fostering for kinship reasons as well as for social advancement and alliance building. The hierarchical structure of the kin or clan system in Buganda has led to a long tradition of alliance-fostering with foster-parents often selected for purposes of enhancing the social position of the child and their birth family. Factors such as potential advantage from acquisition of skills through religious training, occupational apprenticeship or service to a notable family (such as the royal family or their appointed leaders in Buganda) were also considered in the decision-making process on fostering (Roscoe, 1965; Seeley, 2014; Southwold, 1961).

The patrilineal descent system of the Ganda does not mean that a father and his children, once they have grown up, live together. Nahemow (1979) writing before the HIV-epidemic affected the region, explains that families have often been residentially segregated by considerable distances in order to make use of the availability of land. The management of orphans and vulnerable children as a result of the HIV epidemic draws on the variability in family types which resulted from this loose patrilineal structure. The high rates of marital instability (Doyle, 2013; Nabaitu et al., 1994), fostering and residential mobility for work/access to land (Richards, 1973) contributed to ‘considerable variability in the living arrangements and patterns of residential proximity among the Baganda' (Nahemow, 1979: 173). The coming of the HIV epidemic may have increased the variability in the geographical distribution of family members but probably did not alter local co-residence patterns among Baganda kinship groups, bound by allegiances to their patrilineal clan.

### 2.2 Sampling and data collection

This study was nested within a study of household livelihood trajectories (hereafter called the “Trajectories Study”) which was itself based within a larger General Population Cohort (GPC) study, a cohort of 5000 households from 25 villages which has been followed for 24 years (Asiki et al., 2013). The 72 household which included someone who was HIV-positive and 72 matched households where all members were HIV-negative included in the Trajectories study, were purposively selected from the earliest recruited 15 GPC study villages on the basis of whether or not at least one person in the household had tested HIV-positive, and on socio-economic status. Cases (foster-children) were defined as children resident in the 144 households participating in the trajectories study whose closest relation in the household was coded as “grandparent”, “aunt/uncle” or “other relative”. Foster-children recruited for this study were limited to those aged 13–17 years, in line with the ethics committee clearance for the study. In order to avoid possible inclusion of child domestic workers, children recorded as not being related to any household members were excluded from the study. Both households with known HIV infection and those without were included, allowing formative exploration of the ways that HIV may have affected fostering.

The fieldwork, conducted by the first author and a female research assistant, took place from August–November 2006. Heads of household and their spouses were recruited upon securing their consent and the assent of the foster-children. These interviews were used to identify other key individuals (secondary respondents) that had been involved in or left out of the fostering decision-making process. These individuals were then traced based on information provided by the various respondents and, if based in GPC villages, using existing GPC area maps to locate their households. An attempt was made to identify and trace all the key actors central to the fostering decision-making process for each case.

In-depth interviews, lasting between 25 and 40 min were conducted in Luganda. Respondents were visited in their homes. Fifteen eligible cases (foster-children) were identified out of a total of 78 children aged 13–17 years who were living in the 144 trajectories study households (20.5%). One case refused to participate in the study and another child-foster-parent pair was not reached to secure consent and assent despite repeated follow up and thus was not recruited to the study. Data were collected through 48 individual in-depth interviews and two group interviews with foster-children and family members to develop detailed case studies relating to the 13 participating foster-children (mean 3 [range 2–6] key actors/child). Of the 48 individual interviews (30 with females, 18 with males), 29 were with primary respondents (13 foster-children, 16 heads of household and/or their spouses within their foster households) and 19 were among secondary respondents (sending family and extended family members). All but one of the individual and group interviews were recorded and stored digitally with the respondent's consent.

### 2.3 Analysis

A separate interview guide, made up of two sections, was developed for each of the four categories of respondent: foster-child, foster family, sending family, and extended family. The first section covered basic respondent and household profile information and the second covered information on seven key areas: child's background, child's relationship to sending and foster household, foster family household, external perceptions and social position of parents and foster family, fostering decision, links to, and the role of, the extended family in supporting fostering, child's integration into, and roles within, the community and household.

This information was used to examine the structure and strength of kinship, marriage and the inheritance system and the social and political complexity in relation to each case and to enable the study of links between these and HIV. All analysis was then done manually based on the records and transcripts. The interview records were coded and summarised in three key matrices.

1. Attributes or descriptive codes for each respondent
2. Summary matrix showing key information provided by all respondents for each topic (overarching issues discussed) and analytical code (perspectives within topic areas)
3. Case summary highlighting each child's fostering history and current fostering arrangement

For each case, a kinship diagram was drawn to highlight relations both alive and deceased and was used in analysis of information provided on the alternatives considered or rejected for the child's foster care.

### 2.4 Ethical issues

The consent and assent procedures for each child invited into this fostering study were initiated after the household had consented to participate in the Trajectories Study. Prior to interview, each study respondent was invited to listen to information about the study and requested to provide written consent (and assent in the case of children). Given the sensitivity of the topic for some of those interviewed, it was essential that both adults and children had time to get to know and trust the research team. Hence, the researchers were careful to ensure that adequate time was given to the discussions. Ethical approval was provided by the Uganda National Council for Science and Technology (UNCST), the Science and Ethics Committee of the Uganda Virus Research Institute (UVRI) and the London School of Hygiene and Tropical Medicine.

## 3 Findings

The fostering process was documented for a total of 13 children (cases) and 15 foster-parents (Table 2). Nine of the 13 children were fostered by older people in the age range 60–80 years (cases – 5, 6, 7–13) and four children were fostered by younger adults (<60 years) in their immediate or extended family. Ten of the foster-children lived in female-headed households and seven of these households were headed by grandmothers. Eight of the foster-children studied were single orphans (3) or non-orphans (5) and had, in theory, the potential to live with a surviving parent. Four of these children had been fostered by grandparents and one by great-grandparents and the number of children under 18 in each of these homes ranged from 3 to 10.

We order our findings around the themes drawn from life-course analysis: linked lives; social timing; and historical time/human agency (recognising that some degree of overlap exists across the themes).

### 3.1 Linked lives: caregiving and reciprocity

The main role played by older people in this study was that of carer or foster-parent. Many of these older foster-parents explained that, in their view, they had no choice but to take on the responsibility:

*If they [the rest of the family] are not prepared to take care of him, should I throw him away too? [… ] I must stay with him. After all, my role is simply to nurture him. When God gives you a child and that child grows until he is able to look after himself … Is there anything more one would wish for?*

(Grandmother and previous foster-parent, extended family)

Many of the younger relatives had either died or moved to other areas in search of paid employment. Those that had moved to urban areas valued the stability of care that living with grandparents offered their children in contrast to their own lifestyles, and found this arrangement more economical. Experience in parenting, an important factor in the selection of possible foster-parents, played heavily in the favour of grandparents and was explicitly mentioned by 3 sets of parents.

As noted in our introduction, the link between the grandparent and the child was used to ensure the welfare of the grandparent as well as the foster-child. Some older caregivers had been active in seeking out or retaining the caregiving role, as they anticipated significant immediate and future benefits to them from providing that support. This was so in case 6 (an unmarried maternal grandmother fostering her young married daughter's children), case 7 (a single paternal grandmother fostering her married son's child born out of wedlock), and case 8 (great-grandparents fostering their grandson's child born out of wedlock). In all three cases the children were fostered before they were three years old; a decision related to the young age of their parents. The grandparents argued that their children were ill-equipped to be able to provide for and raise the grand-children. As the grand-children and their parents grew older, the children stayed on in the care of these older relatives. The rationale for the extended period of fostering was a combination of the convenience for the parents, who had either married other partners (cases 7 and 8) or had taken up urban employment (case 6), and practical benefits for the ageing foster-parent who needed companionship and support with daily chores.

In the case of a 17 year-old non-orphan (case 7), the grandmother explained why she had decided to accept the responsibility of fostering:

*One must always have old age in mind. You might refuse the child if you still have money, but when the money is nearly all gone and you’ve grown weak, you will have to go and kneel down and ask: “Father, please give me that child to help care for me”. And he will say “You refused the child!” So we take them in and suffer with them. We die with them*.

(Paternal Grandmother, foster-mother)

The importance of fostering as a means of ensuring continued care for relatives as they grew older was a recurrent theme in the dialogue with extended family members. As one paternal uncle explained his family went out of their way to ensure that their ageing mother always had several foster-children in her household:

*You see, I have grandchildren of my own here and we [the family] cannot help [my mother] more than those [foster] children. In fact we make sure we give her children and lots of children have grown up in her care. She started with our own. Now she has cared for nearly all her grandchildren and she has started to care for her great-grandchildren. Yes! She absolutely has to have foster-children who will care for her.*

One parent explained the influence of his mother's ageing on his decision to “give” her his child as follows:

*I was not able to care for my child at the time, so I gave him to his grandmother. When he would grow a bit older he could serve her and fetch her water. I was confident that the child would be raised in able-hands. [… ] The only thing now is that she is weak but she can still look after him even so. (Father, sending family)*

For others, the ageing of grandparents who acted as foster-parents was a concern (recognising that the parental role at their time of life placed a strain upon them). One paternal aunt said:

*I could see the way they [grandparents] were. They had other [foster] children, so I asked [my father] for the child and he gave her to me. Besides, the child's own father had said this to me before he died: “I am leaving this child to you because of the way you have looked after her as she was growing.”*

(Paternal aunt, foster-mother)

### 3.2 Social timing and the challenges faced by older foster-parents

Fostering took a physical toll on some older caregivers, who had, as Appleton (2000) observes expected to be resting at their age. One 60 year-old grandmother commented:

*Fostering is a heavy responsibility and I suffer a lot with this child. We have very little to eat. In fact we farm for our food, but at the moment our [banana] plantations have died. We grow maize, cassava, sweet potatoes and sometimes take some of our harvest into the village to try and earn a bit of money so we can buy some maize meal for the children to eat. I go with my little grandchildren to dig for other families so that we can feed the younger ones. Yes, we suffer.*

(Grandmother, foster-mother)

Another grandmother explained that the burden was not just financial and physical but emotional too. She expressed grief in watching her foster son grow up without the knowledge of any reliable support from his extended family, and this was made worse for her by her own knowledge of the fact that she would soon die and leave him without assistance.

A difficulty for many foster-parents, whatever their age, was that they were expected to bear the full responsibility of providing for the foster-children, and in all the cases studied did so with only occasional support from visiting family members. Where material support was provided towards foster care, it was very often in the form of gifts brought during an occasional or rare visit and included books, some clothing or money towards various school costs such as school meals and examinations. Echoing the words of many of the other grandmothers in the study, one 70 year-old grandmother explained that she had found the entire experience of fostering difficult but particularly the aspect of providing for payments requested by the children's schools. As the children had grown older, however, she explained that they were able to help her more by taking on odd jobs within the neighbourhood and earning some money to help buy their own clothes. The experiences reported by elderly relatives in providing for the foster-children contrasted with the experience reported by younger foster-parents. These foster-parents too had to clothe, nurse, guide, feed and educate the children in their care but they tended to discuss these challenges with a greater sense of confidence in their ability to provide for the children. Many foster-parents were both resolved and resigned to provide for the foster-children through their own means and recognized that these were limited but felt it equally important that they were able to consistently provide the children with emotional care, encouragement and guidance as they grew up.

An additional challenge noted was the relatively limited contact with other relatives due to the lack of mobility and resources of their older caregivers and the stress that this separation had on the children's relationship with those other relatives. Older caregivers were often so concerned that the children did not fall under negative behavioural influences that they imposed considerable restrictions on the children's mobility. This style of parenting was largely in contrast with the younger foster carers who encouraged the children to participate in activities, including local church choirs, village football matches, and public viewing of televised football games. Related to this, older caregivers were not comfortable talking to the children directly about sex, sexual development and HIV. They understood the importance of these discussions, particularly at this stage of the children's lives, but they remained uncomfortable about it, so simply did not touch on the subject in their conversations with the children. Culturally, guidance on sexuality and adolescence are the responsibility of paternal aunts (ssenga) and not grandmothers or parents (Muyinda et al., 2001).

HIV impacted on the fostering process in a number of important ways. While HIV-related stigma related to the child of a person who was thought to have died of AIDS was not mentioned as having directly affected fostering decisions, it was apparent that HIV-related illness and death had led to an increase in the number of children who needed to be fostered and had also reduced the pool of potential foster-parents. Also, HIV was often a source of conflict and eventual collapse of relationships between families, with all relatives associated with the estranged kin ruled out of the fostering decision.

HIV often appeared to have led to a decrease in financial resources, as additional responsibility of caring for other family members meant less financial support for both the foster-parent and the child. Ageing parents constantly spoke of the impact that HIV-related deaths had had on them as they now faced a reality in which they would live as older primary caregivers with little support from the children on whom they had hoped to rely at this time. The decrease in support from the extended family was felt to be particularly important for those living with older relatives who were less able to provide for their own and the foster-child’s financial and material needs. Many of the younger adult relatives that were still alive did not provide regular support. And older foster-parents were reluctant to demand assistance, arguing that these relatives were fully aware of the weight of the fostering responsibility and were likely to be unable or unwilling to help due to responsibilities they themselves had taken on. One grandmother explained her own view of the impact of HIV on her family with both anger and sadness as she thought of her children who had died, reportedly because of AIDS:

*Yes, it affected us; do you think it left us anything positive? [Angry tone then a pause as she recomposes herself] … It affected us. Can I see my children? … [Sad laughter] …. Can I see them? … Yes it affected us …. If you start talking about who has helped and who hasn't you gain a bad reputation. Sometimes honestly, they [relatives] just don't have anything left to give. You can't force them to give you what they don't have*.

(Grandmother, extended family)

All the older foster-parents had been forced to approach traditional and medical health workers and facilities to seek services for the children when they were ill, and had had to play a lead role in helping the young children learn how to seek these services themselves. The return to these roles as the primary caregiver was a challenge for many but it was not new, nor was it something that these older family members would necessarily reject. Their involvement in these social roles and spaces had, in many ways, given many of them not only companionship and support in their households but had helped these older foster-parents find their place in new social networks. These increased responsibilities further enhanced the worth and authority they had in the family and community on top of their traditional position of respect and authority that came from being an “elder”.

### 3.3 Human agency and historical time: older people as advisors, mediators and gatekeepers

While the last 20–25 years of the older carers' lives had been lived during the HIV epidemic, prior to that, all had lived through social and political turmoil during the times of Obote and Idi Amin. This experience was often valued by their younger relatives together with the high regard in the Ganda culture for people who had achieved old age. Even when they did not serve as foster carers, the views of older people in relation to the fostering decision were generally held in very high regard. Six of the children, four of them orphans (1 maternal, 2 paternal, 1 double) and two non-orphans, had been fostered out by grandparents to other people. In the two non-orphan cases, the parents were completely uninvolved in the care of the children and the grandparents had assumed a primary decision-making role. The other seven children in this study had been fostered out by their parents.

In several cases, while they were not selected as foster-parents, older relatives were among the key people consulted or involved in the fostering decision. In case 1, when the young boy ran away from his maternal grandmother's home to a maternal uncle's home nearby, reportedly because he preferred to live with his cousins who were of his age, his grandmother was consulted to request her approval that the child remain in his uncle's care. In case 2, the young girl was fostered out from her paternal grandparents' home to her paternal aunt's home but her paternal grandparents' were viewed by the child, her foster-parents and other relatives as having been the most influential actors in that decision-making process. Both the child and her current foster-parents considered that these grandparents would be the most influential persons in decisions regarding any future movement of the child.

Many grandparents enjoyed privileged access to relatives at all levels and on all sides of the extended family, as theirs was a position of seniority that was respected regardless of lineage affiliation. This seniority frequently enabled them to play a special advisory role or to serve as a crucial link between relatives, mediating or gate-keeping to protect the interests of the child. In ten of the thirteen cases, the foster-child’s parents had either been separated or had never married. In 9 of these cases, tensions and lack of communication between maternal and paternal relatives meant that the side of the family that had not been involved in the care of the child (usually the maternal relatives) had been left out of subsequent negotiations around care arrangements. In two of these cases, the children's grandmothers (one maternal, one paternal), who were also their foster-parents, had played an active role in bridging the divide between the child and their estranged kin. In case 1, while the child was in the care of his maternal grandmother, the grandmother had actively sought out the child's paternal relatives on a regular basis without the knowledge of the other maternal relatives. She relayed news of the child's progress to his otherwise-estranged paternal relatives, ensuring there was a bridge to possible future relationships between the child and his paternal kin. In case 7, the paternal grandmother had recently sought out her foster-child’s estranged mother and arranged for the child to spend a few days with his mother for the first time since he had been separated from her in his infancy.

The parents of five orphans were reported as having taken deliberate steps to prepare for the children's care after their death. For three of them, that preparation came in the form of communication about their preferences with different members of the extended family and the prospective foster-parents themselves. Notably, in all these three cases, grandparents were among those that were informed of the parents' preferences related to what should happen to their child after they died, and asked to provide oversight in the care of the child following the parent's death. The advantage of this strategy was that there was a commitment among multiple family members to protect and provide for these children should the need arise. In addition, grandparents provided parents with a sense of reassurance in this process as they were often seen as trustworthy and sure to keep the child's best interest above all other concerns in the absence of a parent.

This kind of clear succession planning was also present for another boy aged 15 years (case 13) whose parents left their sons in the care of their paternal grandmother in a house that the parents had struggled to build and complete prior to their deaths. They did this to ensure that the children and their ailing grandmother would be secure and sheltered and that, by having occupied the house with their grandmother before their parents' death, the children would not be in danger of falling victim to property grabbers or of losing the property to other relatives. Protection of property was seen as motivation for fostering in another family (case 4) where a child was fostered out by a grandmother into the household of one of her deceased sons to live with a woman widowed when another of her sons died. The child's presence provided the grandmother with an entry point to continue close oversight of her late son's property.

## 4 Discussion

In rural Uganda, older people play a very important role in fostering as caregivers, advisers, mediators and gatekeepers, and they do so with a level of authority over children who are regarded as important resources within the extended family. Conversely, fostering is used by older people as a strategy to ensure both their immediate and future security. In the absence of an effective system of external support for older people from the state or other official organizations, fostering provides the extended family with an opportunity to ensure continued provision of physical and emotional care for elderly relatives as has been described elsewhere (Alber, 2004; Rwezaura, 1989; Schroder-Butterfill, 2004). The emotional and many practical benefits to both older people and the children that they foster are important and should not be discounted.

Nevertheless, the return to the role of primary caregiver, a role all would have expected to have left behind, was a challenge for many older people. As has been previously reported, they may face periods of loneliness, despondency and depression as the burden of caring for their grandchildren, great nephews and nieces, etc. was coupled with grieving for relatives lost to HIV-related deaths (Dolbin-MacNab, 2006; Freeman and Nkomo, 2006; McKinnon et al., 2013; Wright et al., 2012). As others have also reported, our study found that being fostered by an older carer could also be a challenge for the child, who may face a heavy care burden as well as a loss of educational and social opportunities (Dalen et al., 2009; Morantz et al., 2013; Schenk et al., 2010; Skovdal et al., 2009). Yet, this role was not something that these older family members would necessarily reject or even regret. In central Uganda, great importance is placed on societal regeneration based on the transmission of values through parenting carried out through the extended family (Foster, 2000; Madhavan, 2004; Page, 1989; Richter et al., 2009); grandparents have great authority over this function.

The extended family operated quite effectively as a safety net ensuring care was provided for orphans, often as had been explicitly desired by the parents prior to their death (Mathambo and Gibbs, 2009; Seeley, 2014). Older people in the case studies reported in this paper continued to enjoy a high degree of influence within the family despite the relative increase in monetary wealth and access to opportunities for many of the young adults in the family. Older people, as home and land owners, represented both stability of residence as well as success in terms of having lived to an old age, and often gained even further respect and influence in the extended family through taking on the responsibility of foster-child care. The authority shown by older people and the respect for this authority in fostering decisions and care reflects the continued significance of the role of older people in providing counsel on, and sometimes exercising control over, family resources. The value placed on the views of older people in relation to the fostering decision are a reflection of the traditional order of social organization in parts of Africa that Rwezaura (1989) described, in which older people occupied an enviable position of control over such strategic resources as children, on which the extended family was dependent for social stature, productivity and survival.

From the point of view of carers in this study, HIV had definitely led to an increased level and a new range of responsibilities for older people. On the positive side, as demonstrated, this has led to a renewed respect for and recognition of older people and their influence in families and in the communities. The added responsibilities present challenges to older people that could be addressed through specific interventions, perhaps through community based groups, designed to help them manage these roles.

A 1995 study on orphan care in 6 districts from the west to the east of Uganda observed that a key gap and opportunity in the response to care of orphans and vulnerable children through fostering existed in the social work system (Ntozi and Mukiza-Gapere, 1995). That study showed that the social work system was inadequately developed and so was underutilized in providing critical support to families in their fostering decision-making processes as well as in the areas of follow-up to ensure quality of care and support to children and their carers in foster homes, and effective management of information by the state for planning of resources to support families. Their observations remain relevant to the situations and accounts of the families studied here in Kyamulibwa over 10 years after their study.

An important strength of this study was its ability to carefully document the viewpoints of a wide-variety of actors in the fostering process. In focusing on older people, this paper was able to provide a unique insight into their role in the fostering process in the context of the HIV epidemic. Situating the study within the existing General Population Cohort study allowed the identification of a representative sample of all fostered children aged 13–17 years living with a relative in the study area.

This study was designed to provide in-depth understanding around fostering decisions. The case study approach focused on a limited number of children. While this approach is likely to have been effective in providing a comprehensive understanding of the dynamics affecting fostering decisions based on processes that had successfully resulted in a child being fostered by a relative, it is important to note that the small sample, the study settings within rural Kyamulibwa and the reliance on GPC data that identified “fostered” children may limit the generalisability of the findings. For example, it does not tell us anything about children who had moved to urban areas. Even so, the fostering experiences described by people in this study are very likely to be similar in many other patrilineal rural settings in Uganda because of the important common factors of patrilineal social organization, limited access to services and resources within the extended family in rural areas, and the extended impact on families of the HIV epidemic (loss of parents, other adults and orphaning). Fostering decisions in urban settings on the other hand, are likely to be different, as there is a considerable difference in access to resources affecting both the motivations of sending and receiving families with potentially great effect on the profile of foster-parents and foster-children, and the role of maternal and paternal relatives. One might speculate, for example, that non-relative and alliance fostering, and the occurrences of “street children” who are either not fostered or have run away from their foster homes, might be more common in urban settings. The study, by design, had not catered for examination of motivations for fostering of non-relatives. The motivations for fostering among non-relatives may have been different as might have been the success of these arrangements in terms of duration of fostering. The viewpoints and coping mechanisms of the foster-children, shown by others to be an important dimension to explore (Skovdal et al., 2009), were not presented in this paper, but we plan to focus on these in a further report.

This research relied on the strategy of saturation of informants from an extended family in order to build case studies. The sensitivity of the research topic might have affected access to information and respondents in the study as some might have feared social judgement or wanted to avoid tensions in the family. The study team did, however, manage to secure consent from 13 of the 15 eligible families identified through the trajectories study and within each of the families we also managed to reach and interview all relevant respondents from the extended family, who were traceable and within a reasonable distance.

## 5 Conclusions

In the study area the extended family system continues, in the majority of cases, to provide care for all children within the family, and, through fostering, continues to provide this care to children made vulnerable by HIV or by other circumstances. Nevertheless, urgent efforts and investment are required to complement these community and family resources with comprehensive and well-managed social protection and welfare programmes. The aim would be to strengthen families' ability to manage. However, recognizing that vulnerability is widespread and resources limited, these programmes should prioritize the community's poorest households, and particularly those with children and older people but no younger adults.

## Figures and Tables

**Table 1:** Distribution and profile of children (aged 0–17 years) in foster care in Uganda in 2011.

	Total number of children	Proportion of children fostered (Number)	Profile of children in foster care^a^			
			Non-orphans (%)	Orphans		
				Maternal (%)	Paternal (%)	Double (%)
**Age**						
0–4	8361	9.1% (761)	85.7	4.4	6.6	3.3
5–9	7688	18.9% (1453)	70.4	8.5	13.8	7.4
10–14	6659	25.3% (1685)	61.7	9.1	16.6	12.6
15–17	2875	29.7% (854)	60.3	8.4	16.2	14.8
**Sex**						
Male	12,947	17.4% (2253)	66.7	8.6	13.8	10.9
Female	12,636	19.8% (2502)	69.2	8.1	14.6	8.6
**Residence**						
Urban	3058	22.5% (688)	67.1	8.0	16.4	8.4
Rural	22,525	18.1% (4077)	68.0	8.3	13.8	9.9
Total	25,583	18.6% (4758)	67.7	8.1	14.0	9.7

a Includes children with missing information on their parents' survival status so percentages will not add up to 100%.

**Table 2:** Case and foster parent profiles.

Case	Child's age	Sex	Orphanhood status	Duration in current foster home	Age at fostering into current household	Previous foster homes	Age at 1st fostering	Relationship to Foster parent(s)	Age of foster parent(s)	Occupation
1	13	M	Maternal	5 Months	13 years	2 (Stepmother, maternal grandmother)	5 years	Maternal uncle	45	Farmer/Retired community leader
2	14	F	Double	5 Years	9 years	2 (Paternal grandparents, paternal uncle)	2 years	Paternal aunt (Ssenga)	32	Farmer
3	13	F	Paternal	5 Years	8 years	1 (Paternal grandparents)	6 years	Paternal aunt (Ssenga)	37	Farmer
4	15	F	Double	6 Years	9 years	2 (Paternal aunt, paternal grandmother)	5 years	Female in-law (deceased paternal uncle's wife)	45	Tailor/Farmer
5	15	M	Double	12 years	3 years	0	3 years	Paternal grandmother's sister	68	Farmer
6	16	F	Non-orphan	14 years	2 years	0	2 years	Maternal grandmother	60	Farmer
7	17	M	Non-orphan	16 years	1 year	0	1 year	Paternal grandmother	60	Farmer
8	14	F	Non-orphan	12 years	2 years	0	2 years	Paternal great grandfather and great grandmother	80/73	Farmers
9	14	M	Paternal	8 months	12 years	1 (Maternal grandparents)	13 years	Paternal grandmother	70	Farmer
10	14	M	Non-orphan	6 years	8 years	1 (Maternal grandparents)	2 years	Paternal grandmother	65	Farmer
11	13	M	Non-orphan	6 years	7 years	1 (Paternal grandparent)	7 years	Paternal grandfather and grandmother	69/63	Farmers
12	14	F	Double	8 years	6 years	0	6 years	Paternal grandmother	67	Farmer
13	15	M	Double	4 years	11 years	0	11 years	Paternal grandmother	78	Farmer
